# Overweight, Obesity, and Late-Life Sarcopenia Among Men With Cardiovascular Disease, Israel

**DOI:** 10.5888/pcd17.200167

**Published:** 2020-12-24

**Authors:** Miri Lutski, Galit Weinstein, David Tanne, Uri Goldbourt

**Affiliations:** 1 The Israel Center for Disease Control, Israel Ministry of Health, Tel Hashomer, Israel; 2 School of Public Health, Faculty of Social Welfare and Health Sciences, University of Haifa, Haifa, Israel; 3 Department of Neurology, Sackler Faculty of Medicine, Tel Aviv University, Tel Aviv, Israel; 4 Stroke and Cognition Institute, Rambam Health Care Campus, Haifa, Israel; 5 Department of Epidemiology and Preventive Medicine, School of Public Health, Sackler Faculty of Medicine, Tel Aviv University, Tel Aviv, Israel

## Abstract

**Introduction:**

Little is known about the association between obesity and sarcopenia — age-related loss of muscle mass and function — among patients with cardiovascular disease. We investigated the association between overweight, obesity, and sarcopenia among community-dwelling men in Israel with cardiovascular disease.

**Methods:**

A subset of 337 men (mean age at baseline 56.7 [SD, 6.5]) who previously (1990–1997) participated in the Bezafibrate Infarction Prevention trial underwent a neurovascular evaluation as part of the Bezafibrate Infarction Prevention Neurocognitive Study 15.0 (SD, 3.0) years after baseline and a sarcopenia evaluation 19.9 (SD, 1.0) years after baseline. We applied a multinomial logistic model to estimate odds ratios and 95% CIs for 3 categories of sarcopenia: no evidence of sarcopenia (ie, robust), probable sarcopenia, and sarcopenia.

**Results:**

We found sarcopenia among 54.3% of participants with obesity (body mass index [BMI, in kg/m^2^] ≥30.0), 37.0% of participants who were overweight (25.0 ≤ BMI ≤29.9), and 24.8% of participants with normal weight (BMI 18.5 to 24.9). In a comparison of BMI ≥25.0 and BMI <25.0, adjusting for covariates, the odds ratio of having probable sarcopenia was 3.27 (95% CI, 1.68–6.36) and having sarcopenia was 5.31 (95% CI, 2.50–11.27).

**Conclusion:**

We found a positive association between obesity and late-life sarcopenia and suggest that obesity might be an important modifiable risk factor related to sarcopenia among men with cardiovascular disease.

**Summary**

**What is known about this topic?**

Cardiovascular disease (CVD) might accelerate the development of sarcopenia. However, little is known about the association between obesity and sarcopenia among patients with CVD.

**What is added by this report?**

We observed a positive association between men who were overweight or obese and had CVD and late-life sarcopenia.

**What are the implications for public health?**

Given the growth of the population aged 65 or older and the high prevalence of obesity and CVD, it is important to identify people with CVD and potentially preventable risk factors for sarcopenia.

**MEDSCAPE CME**

In support of improving patient care, this activity has been planned and implemented by Medscape, LLC and *Preventing Chronic Disease*. Medscape, LLC is jointly accredited by the Accreditation Council for Continuing Medical Education (ACCME), the Accreditation Council for Pharmacy Education (ACPE), and the American Nurses Credentialing Center (ANCC), to provide continuing education for the healthcare team.

Medscape, LLC designates this Journal-based CME activity for a maximum of 1.00 AMA PRA Category 1 Credit(s)™. Physicians should claim only the credit commensurate with the extent of their participation in the activity.

Successful completion of this CME activity, which includes participation in the evaluation component, enables the participant to earn up to 1.0 MOC points in the American Board of Internal Medicine’s (ABIM) Maintenance of Certification (MOC) program. Participants will earn MOC points equivalent to the amount of CME credits claimed for the activity. It is the CME activity provider’s responsibility to submit participant completion information to ACCME for the purpose of granting ABIM MOC credit.

**Release date: December 24, 2020; Expiration date: December 24, 2021**

**Learning Objectives**

Upon completion of this activity, participants will be able to:

1. Describe the prevalence of sarcopenia and associated factors among community-dwelling elderly men with CVD
2. Assess increased risk for sarcopenia as a function of body mass index among community-dwelling elderly men with CVD
3. Identify clinical and public health implications of the association among overweight, obesity, and late-life sarcopenia among community-dwelling elderly men with CVD

**EDITOR**

Ellen Taratus, MS, ELS

Editor, *Preventing Chronic Disease*

Disclosure: Ellen Taratus has disclosed no relevant financial relationships.

**CME AUTHOR**

Laurie Barclay, MD

Freelance writer and reviewer

Medscape, LLC

Disclosure: Laurie Barclay, MD, has disclosed no relevant financial relationships.

**AUTHORS**

Miri Lutski, PhD

The Israel Center for Disease Control

Israel Ministry of Health

Tel Aviv, Israel

Disclosure: Miri Lutski, PhD, has disclosed no relevant financial relationships.

Galit Weinstein, PhD

School of Public Health

Faculty of Social Welfare and Health Sciences

University of Haifa

Haifa, Israel

Disclosure: Galit Weinstein, PhD, has disclosed no relevant financial relationships.

David Tanne, MD

Department of Neurology

Sackler Faculty of Medicine

Tel Aviv University

Tel Aviv, Israel

Stroke and Cognition Institute

Rambam Health Care Campus

Haifa, Israel

Disclosure: David Tanne, MD, has disclosed no relevant financial relationships.

Uri Goldbourt, PhD

Department of Epidemiology and Preventive Medicine

School of Public Health

Sackler Faculty of Medicine

Tel Aviv University

Tel Aviv, Israel

Disclosure: Uri Goldbourt, PhD, has disclosed no relevant financial relationships.

## Introduction

Sarcopenia, from the Greek “poverty of flesh,” is a highly prevalent geriatric syndrome first described by Rosenberg in 1989 as the age-related loss of muscle mass and function (1). Accumulating evidence suggests that sarcopenia is associated with adverse health outcomes such as frailty, falls, disability, admission to nursing homes, and mortality (2). Several underlying mechanisms are linked with the development of sarcopenia, including impaired neuromuscular function, hormonal changes, increased inflammation, changes in body-fat distribution, poor nutritional status, and various chronic conditions, yet not all have been fully elucidated (3). The most studied approach in modifying risk factors for sarcopenia is resistance exercise. Numerous treatments of sarcopenia, including protein supplementation and pharmacological interventions, have limited value (4).

Obesity-mediated factors may aggravate sarcopenia in older people and maximize its effects on physical disability, morbidity, and mortality (5,6). Several studies investigated the association of obesity with sarcopenia (7–10). Findings on the association between overweight and sarcopenia are controversial (7). The prevalence of cardiovascular disease (CVD) in middle and old age is increasing, partly as a result of increases in the prevalence of obesity (11,12). Furthermore, CVD might accelerate the development of sarcopenia, and both have been strongly tied to chronic low-grade inflammation, insulin resistance, and obesity (13). However, little is known about the association between obesity and sarcopenia in patients with CVD. The aim of this study was to describe the association between overweight, obesity, and late-life sarcopenia among community-dwelling men aged 64 or older with CVD.

## Methods

Our study sample consisted of a subset of patients from 8 hospitals who resided in the central region of Israel and who previously participated in the Bezafibrate Infarction Prevention (BIP) clinical trial of lipid modification during 1990–1997 (N = 1,232) and then in the BIP Neurocognitive Study during 2004–2013. The study design and procedures of the BIP trial are detailed elsewhere (14). In brief, the BIP study was a placebo-controlled randomized clinical trial investigating the efficacy of a 400-mg daily dose of bezafibrate, a fibric derivative, in secondary prevention among patients with established stable coronary heart disease. To be included in our study, the lipid profile of patients had to fall within these parameters: serum total cholesterol 180 to 250 mg/dL, low-density lipoprotein cholesterol ≤180 mg/dL (≤160 mg/dL for people aged <50), high-density lipoprotein cholesterol ≤45 mg/dL, and triglycerides ≤300 mg/dL. Other exclusion criteria were renal failure, defined as a serum creatinine level ≥1.5 mg/dl or nephrotic syndrome; liver failure, defined as serum glutamate pyruvatetransominase >60 U/L; and stroke, assessed by reviewing records from hospital or emergency department discharge, a primary care physician, or a neurologist (14).

The BIP Neurocognitive Study consisted of 2 follow-up evaluations (15). The first follow-up evaluation (time 1; n = 546) was performed during 2004–2009, an average of 15.0 (SD, 3.0) years after recruitment to BIP; it assessed neurovascular and cognitive function. Patients were re-examined during 2011–2013 (time 2; n = 351), 19.9 (SD, 1.0) years after recruitment; this examination assessed sarcopenia and re-assessed cognitive function. The mean interval between time 1 and time 2 was 4.8 (SD, 1.3) years. Patients were assessed at a central research center (the Sagol Neuroscience Center, Sheba Medical Center, Ramar Gan, Israel), or if a patient was unable or unwilling to attend the medical center, the assessment occurred at their residence.

The institutional review boards of the Sheba Medical Center Ethics Committee approved the study and informed consent was obtained from all participants.

### Baseline measurements (1990–1993)

Methods for baseline assessment of the BIP study are described elsewhere (16). Height and weight (without shoes) were measured at baseline (1990–1992) using a standard stadiometer and an electronic weighing scale. Body mass index (BMI) was calculated as body weight in kilograms divided by height in meters squared (kg/m^2^). Weight was categorized as normal (BMI 18.5–24.9, hereinafter referred to as BMI <25.0), overweight (BMI 25.0–29.9), or obese (BMI ≥30.0). Blood samples were drawn from each study patient at baseline of the BIP trial. Samples were collected after at least 12 hours of fasting, with the use of standardized equipment and procedures and transferred to a central study laboratory. Insulin resistance was defined as the upper quartile of the homeostatic model assessment of insulin resistance, calculated as fasting insulin (μU/ml) × fasting glucose in mg/dL/405. C-reactive protein concentrations were measured using standard automated procedures with commercially available kits (Roche Diagnostics). Angina severity at baseline of the BIP study was classified according to the Canadian Cardiovascular Society angina classification, the standard measure for grading the severity of effort-induced angina (17). Participants were categorized into 2 groups: 1) no angina pectoris or angina pectoris class 1, and 2) angina pectoris class 2 and above. Physical activity was assessed by asking participants, “Do you participate in one or more of the following activities such as walking, swimming, jogging, gymnastics, biking, dancing, tennis, gardening, gym and rehabilitation activities?” Responses were categorized as any physical activity or sedentary physical activity. Data on education, place of birth, smoking, and comorbidity were collected through a questionnaire at baseline.

### Sarcopenia evaluation (time 2)

Sarcopenia was defined by using the European Working Group on Sarcopenia in Older People definitions of low muscle strength, low muscle mass, and low physical performance (2). Probable sarcopenia was defined as low muscle strength or low muscle mass. Sarcopenia was defined as low muscle strength and low muscle mass and/or low physical performance.

Muscle strength was defined as isometric dominant handgrip strength, which was assessed by using a Jamar hydraulic hand dynamometer (Sammons Preston). Muscle strength was categorized as low if ≤29 kg (for patients with BMI ≤24.0), ≤30 kg (for patients with BMI 24.1–28.0), and ≤32 kg (for patients with BMI >28.0). The test was carried out twice and the higher score was used.

Muscle mass was assessed by bioelectric impedance analysis using a Tanita BC-545 8-contact electrode body composition analyzer. To calculate body composition, the computer software in the bioelectric impedance analysis system uses the measured impedance, the programmed person’s sex and height, and the measured weight. The device measures fat-free mass in kilograms and the percentage of total body fat according to the equation provided by the device’s software. Skeletal muscle mass was calculated by using the following equation: skeletal muscle mass = 0.566 × fat-free mass. Skeletal muscle mass index, adjusted for weight, was calculated as (skeletal muscle mass × 100)/weight (18). Low skeletal muscle mass index was defined as 37.4% or lower (19).

We used gait speed to determine physical performance. Gait speed was measured by gait time in seconds using a 5-meter timed walk test. Usual gait speed of less than 1 meter per second signifies a high risk of health-related outcomes in well-functioning older people (20). The test instructions were as follows: “On the word ‘go’ start walking at your regular pace to the line on the floor.” We used height-adjusted time as the cutoff. Low physical performance was denoted as ≥6 seconds (for height ≤173 cm [68 inches]) and ≥5 seconds (for height >173 cm) (21).

### Additional assessments

In both evaluations (time 1 and time 2), data on comorbidities and hospitalizations, medication use, smoking status, physical activity, and anthropometric measurements were collected systematically. In addition, systolic blood pressure, diastolic blood pressure, and weight and height were measured.

Incident stroke during follow-up was assessed by reviewing records from hospital or emergency department discharge, primary care physicians, or neurologists. We used a score of 5 or more on the 15-item version of the Geriatric Depression Scale (22) to indicate clinically significant depressive symptoms. Patients completed the NeuroTrax computerized cognitive test (NeuroTrax Corporation). A description of this test is available elsewhere (23). All NeuroTrax scores are normalized according to age- and education-specific normative data and scaled to an IQ-style scale with a mean of 100 and SD of 15. Dementia and incident stroke during follow-up were determined by an adjudication committee composed of 3 investigators, 2 of whom were experienced board certified neurologists. A diagnosis of dementia was based on a cognitive evaluation, a clinical interview, and data collected and was in accordance with criteria of the *Diagnostic and Statistical Manual of Mental Disorders, 4th Edition* (24). An occurrence of stroke was determined on the basis of World Health Organization criteria (25).

Cerebrovascular reactivity, a marker of cerebral microvascular function, was evaluated by transcranial Doppler (Trans-Link 9900 [Rimed]), which measures the breath-holding index (26). Participants were categorized into normal (≥0.69) or impaired (<0.69) cerebrovascular reactivity on the basis of the mean breath-holding index of both middle cerebral arteries according to previously established standard parameters (27). Carotid intima-media thickness (cIMT), a measure of cerebral large-vessel atherosclerosis, and plaque presence were measured at the far wall of both common carotid arteries using high-resolution B-mode ultrasound. Participants were classified into the following 2 categories according to previously established standard parameters (28): cIMT ≥0.93 mm (elevated) and/or bilateral carotid plaques or cIMT <0.93 (normal) and without bilateral carotid plaques.

### Statistical analysis

We summarized data on the clinical characteristics of patients as percentage and mean (SD), unless the distribution was strongly skewed, in which case we summarized data as median and interquartile range (IQR). We compared variables between BMI and sarcopenia groups by using analysis of variance or the Kruskal–Wallis test for continuous variables and the χ^2^ test for categorical variables. We used multinomial logistic regression to estimate odds ratios (ORs) and 95% CIs for sarcopenia. The categories of the outcome variable were sarcopenia, probable sarcopenia, and robust (ie, no evidence of sarcopenia); we used robust as the reference category in comparisons. We first adjusted for age, education, and birthplace, then additionally for systolic blood pressure, physical activity, diabetes, insulin resistance, C-reactive protein, high-density lipoprotein cholesterol, and triglycerides. Subsequently, we further adjusted for depressive symptoms (Geriatric Depression Scale score ≥5 vs <5), global cognitive score, and Doppler ultrasound indices of cerebrovascular disease at time 1.

Because of loss to follow-up of eligible patients who had either died or refused participation, we estimated the probability of every person to reach the sarcopenia assessment and calculated the inverse probability weights (29). We compared the results of weighted analysis with nonweighted analysis. Data were analyzed using SPSS version 21 (IBM Corporation).

## Results

Of the 1,232 patients eligible for the evaluation at time 1, 214 had died; 259 refused to participate; 102 could not be contacted; 45 were unable to participate because of dementia, language incompatibility, vision or hearing defects, or physical disability; and 54 were excluded because of missing data. Twelve women were not included because of the small sample. This process of exclusion resulted in 546 men available for evaluation at time 1 (Figure). A total of 351 patients were re-assessed at time 2; of these, 337 had BMI measurements and a sarcopenia evaluation. The attrition from time 1 and time 2 was mainly a result of interim death (n = 114); in addition, 58 refused to participate, 4 could not be contacted, and 19 were unable to participate. The mean (SD) age of the study sample was 56.7 (6.5) at baseline, 71.8 (6.5) at time 1, and 77.1 (6.4) at time 2.

**Figure:**
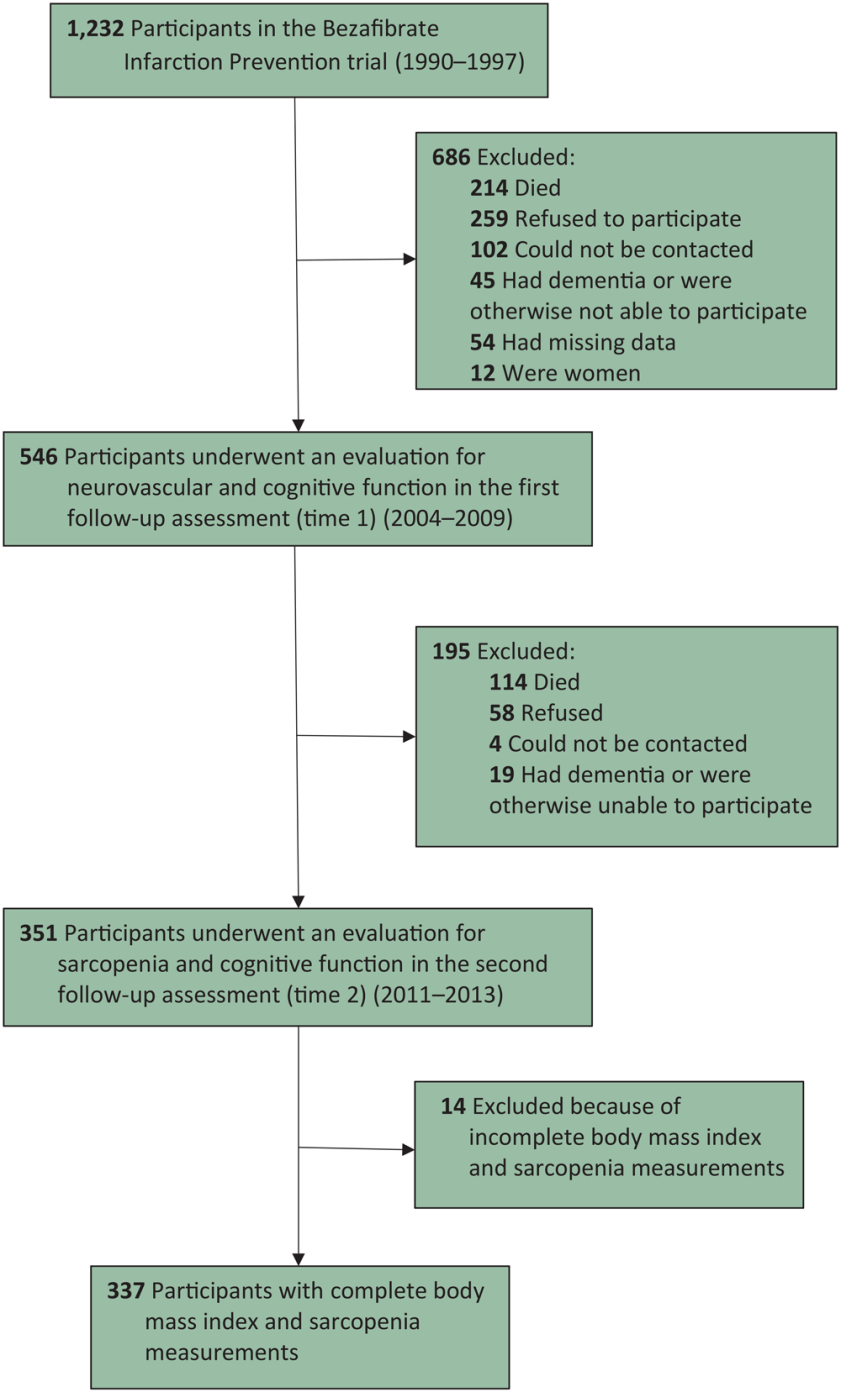
Study flowchart. Participants from 8 hospitals in central Israel were initially recruited for the Bezafibrate Infarction Prevention (BIP) clinical trial of lipid modification during 1990–1997 and were also in the BIP Neurocognitive Study. The BIP Neurocognitive Study consisted of 2 follow-up evaluations: time 1 (2004–2009) and time 2 (2011–2013).

Of the 337 patients, 109 (32.3%) were classified as robust, 112 (33.2%) as probable sarcopenia, and 116 (34.4%) as sarcopenia (Table 1). Sarcopenia was significantly related to older age, less education, more severe angina pectoris (angina pectoris class ≥2), and a higher systolic blood pressure at baseline. In addition, participants with sarcopenia had poorer global cognitive function, more cerebrovascular diseases, and a higher level of depression at time 1 compared with their counterparts.

**Table 1:** Baseline Characteristics of Participants in the Bezafibrate Infarction Prevention Neurocognitive Study, by Sarcopenia Status at Time 2, Israel, 2011–2013

Characteristic	Sarcopenia Status (N = 337)			
	Robust^a^ (n = 109)	Probable Sarcopenia^b^ (n = 112)	Sarcopenia^c^ (n = 116)	*P* Value^d^
**Baseline (1990–1993)**				
**Age, mean (SD), y**	54.0 (5.7)	55.6 (6.4)	59.9 (5.8)	<.001
**<12 Years of education, n (%)**	25 (22.9)	37 (33.0)	43 (37.1)	.02
**Birthplace, n (%)**				
Middle East	32 (29.4)	33 (29.5)	39 (33.6)	.02^e^
Europe	47 (43.1)	46 (41.1)	55 (47.4)	
Israel	30 (27.5)	33 (29.5)	22 (19.0)	
**Smoking, n (%)**				
Currently	5 (4.6)	10 (8.9)	9 (7.8)	.61
Former	75 (68.8)	67 (59.8)	73 (62.9)	
Never	29 (26.6)	35 (31.2)	34 (29.3)	
**Previous myocardial infarction, n (%)**	87 (79.8)	92 (82.1)	87 (75.0)	.40
**Diabetes, n (%)**	10 (9.2)	9 (8.0)	13 (11.2)	.71
**Chronic kidney disease,^f^ n (%)**	26 (23.9)	24 (21.6)	39 (33.6)	.09
**Insulin resistance,^g^ n (%)**	25 (23.6)	32 (30.5)	23 (20.9)	.25
**Angina class,^h^ n (%)**				
≥2	14 (12.8)	14 (12.5)	18 (15.5)	.03^e^
<2	95 (87.2)	98 (87.5)	98 (84.5)	
**Previous hypertension, n (%)**	22 (20.2)	31 (27.7)	41 (35.3)	.04
**Any physical activity, n (%)**	83 (78.3)	80 (72.1)	82 (71.3)	.44
**Blood glucose ≥100 mg/dL, n (%)**	34 (31.2)	33 (29.7)	51 (44.0)	.04
**Body mass index, n (%)**				
18.5 to <25.0 kg/m^2^	57 (52.3)	34 (30.4)	30 (25.9)	<.001
25.0–29.9 kg/m^2^	48 (44.0)	66 (58.9)	67 (57.8)	
≥30.0 kg/m^2^	4 (3.7)	12 (10.7)	19 (16.4)	
**Height, mean (SD), m**	1.7 (0.6)	1.7 (0.7)	1.7 (0.6)	<.001
**Blood pressure, mean (SD), mm Hg**				
Systolic	126 (15)	129 (15)	133 (17)	<.001
Diastolic	80 (9)	80 (8)	81 (8)	.66
**Cholesterol, mean (SD), mg/dL**				
Total	215 (16)	214 (19)	213 (19)	.58
Low-density lipoprotein	150 (16)	149 (17)	150 (18)	.91
High-density lipoprotein	34 (5)	35 (5)	35 (5)	.35
**Triglycerides, median (IQR), mg/dL^i^**	141 (121–184)	135 (108–190)	133 (98–174)	.08
**C-reactive protein, median (IQR), mg/dL^i^**	2.2 (1.1–4.5)	2.1 (1.3–4.0)	2.4 (1.5–4.9)	.23
**Time 1 (2004–2009)**				
Age, mean (SD), y	68.8 (5.4)	70.7 (6.4)	75.3 (5.8)	<.001
Common carotid intima-media thickness, mean (SD), mm	0.93 (0.2)	0.97 (0.2)	0.10 (0.2)	.04
Impaired cerebrovascular reactivity, n (%)	41 (39.4)	33 (32.7)	56 (51.4)	.02
Bilateral carotid plaque, n (%)	49 (45.4)	51 (47.2)	69 (61.6)	.03
Global cognitive score,^j^ mean (SD)	98.8 (8.9)	96.8 (10.0)	95.2 (8.9)	.02
Geriatric Depression Scale,^k^ score ≥5, n (%)	9 (8.3)	17 (15.2)	26 (22.8)	<.001
**Time 2 (2011–2013)**				
Age, mean (SD), y	74.2 (5.5)	76.0 (6.4)	80.4 (5.7)	<.001

Abbreviation: IQR, interquartile range.

a No evidence of sarcopenia.

b Defined as low muscle strength or low muscle mass according to European Working Group on Sarcopenia in Older People (2).

c Defined as low muscle strength and low muscle mass and/or low physical performance according to European Working Group on Sarcopenia in Older People (2).

d *P* value determined by analysis of variance or Kruskal–Wallis test for continuous variables and χ^2^ test for categorical variables, unless otherwise indicated; *P* < .05 considered significant.

e *P* for trend determined by χ^2^ test; *P* < .05 considered significant.

f Defined as estimated glomerular filtration rate <60 mL/min/m^2^.

g Defined as homeostatic model assessment of insulin resistance in the top quartile (≥1.60).

h Classfication according to Canadian Cardiovascular Society angina classification (17); the larger the value, the greater the severity.

i Median (IQR) presented because of nonnormal distribution of data.

j Global cognitive score scaled to an IQ-style scale with mean of 100 and SD of 15. Patients completed the NeuroTrax computerized cognitive test (NeuroTrax Corporation). A description of this test is available elsewhere (23).

k Geriatric Depression Scale (22) from 0 to 15; score of ≥5 indicates clinically significant depressive symptoms.

In general, patients with obesity, compared with patients without obesity, more often had diabetes and insulin resistance, a blood glucose level ≥100 mg/dL, and higher levels of C-reactive protein, and they were less physically active at baseline. We found sarcopenia among 54.3% of patients with obesity, 37.0% of patients who were overweight, and 24.8% of patients with normal weight (*P* for trend <.001).

Adjusting for age, education, birthplace, systolic blood pressure, physical activity, insulin resistance, C-reactive protein, high-density lipid cholesterol, and triglycerides, the estimated adjusted OR (95% CI) for probable sarcopenia for patients with BMI ≥25.0, compared with patients with BMI <25.0, was 2.88 (1.54–5.36) and for sarcopenia was 5.04 (2.51–10.15). Additional adjustment for global cognitive score, Doppler ultrasound indices of cerebrovascular disease, and depressive symptoms did not materially alter the results (Table 2). An increment of 1.0 BMI unit was associated with an adjusted OR (95% CI) of 1.33 (1.16–1.53) for probable sarcopenia and of 1.38 (1.19–1.59) for sarcopenia (Table 3). The adjusted OR (95% CI) for probable sarcopenia for patients with obesity was 6.51 (1.56–27.13) and for patients who were overweight (but not obese) was 3.03 (1.54–5.98). The adjusted OR (95% CI) for sarcopenia for patients with obesity was 13.49 (3.12–58.20) and for patients who were overweight was 4.65 (2.16–10.04).

**Table 2:** Multinomial Logistic Regression for Association Between BMI Groups (≥25.0 vs <25.0) at Baseline (1990–1993) and Sarcopenia Status at Time 2 (2011–2013) Among a Sample of Men (n = 337) Participating in Bezafibrate Infarction Prevention Neurocognitive Study, Israel^a^

Model	Probable Sarcopenia		Sarcopenia	
	OR (95% CI)	*P* Value	OR (95% CI)	*P* Value
**Model 1^b^**				
BMI ≥25	2.95 (1.64–5.29)	<.001	4.94 (2.57–9.48)	<.001
BMI <25	1 [Reference]		1 [Reference]	
**Model 2^c^**				
BMI ≥25	2.88 (1.54–5.36)	.001	5.04 (2.51–10.15)	<.001
BMI <25	1 [Reference]		1 [Reference]	
**Model 3^d^**				
BMI ≥25	3.27 (1.68–6.36)	<.001	5.31 (2.50–11.27)	<.001
BMI <25	1 [Reference]		1 [Reference]	
**Model 4^e^**				
BMI ≥25	2.72 (1.81–4.09)	<.001	4.52 (2.89–7.05)	<.001
BMI <25	1 [Reference]		1 [Reference]	
**Model 5^f^**				
BMI ≥25	3.76 (1.84–7.68)	<.001	7.78 (3.24–18.69)	<.001
BMI <25	1 [Reference]		1 [Reference]	

Abbreviations: BMI, body mass index; OR, odds ratio.

a In all comparisons, reference outcome value is robust, defined as no evidence of sarcopenia. The category BMI <25 excludes underweight (BMI <18.5).

b Model 1 = age, education (≥12 y vs <12 y), and birthplace (Europe, Middle East vs Israel).

c Model 2 = Model 1 + systolic blood pressure (continuous), physical activity, diabetes, insulin resistance (top quartile vs others), C-reactive protein, high-density lipoprotein cholesterol, and triglycerides (continuous).

d Model 3 = Model 2 + impaired cerebrovascular reactivity vs normal, carotid intima-media thickness, global cognitive score, and geriatric depression score ≥5 at time 1 (2004–2009).

e Model 4 = Model 3 applying inverse probability weights.

f Model 5 = Model 3 after excluding 53 participants with stroke and dementia at time 2 (2011–2013).

**Table 3:** Multinomial Logistic Regression for Association Between BMI (as a Continuous Variable) and Sarcopenia Status Among a Sample of Men (n = 337) Participating in Bezafibrate Infarction Prevention Neurocognitive Study, Israel, 2011–2013^a^

Model	Probable Sarcopenia		Sarcopenia	
	OR (95% CI)	*P* Value	OR (95% CI)	*P* Value
Model 1^b^	1.24 (1.11–1.39)	<.001	1.34 (1.19–1.51)	<.001
Model 2^c^	1.28 (1.12–1.45)	<.001	1.39 (1.21–1.59)	<.001
Model 3^d^	1.33 (1.16–1.53)	<.001	1.38 (1.19–1.59)	<.001
Model 4^e^	1.28 (1.18–1.39)	<.001	1.34 (1.23–1.45)	<.001
Model 5^f^	1.33 (1.15–1.54)	<.001	1.42 (1.21–1.66)	<.001

Abbreviations: BMI, body mass index; OR, odds ratio.

a In all comparisons, reference outcome value is robust, defined as no evidence of sarcopenia.

b Model 1 = age, education (≥12 y vs <12 y), birthplace (Europe, Middle East vs Israel).

c Model 2 = Model 1 + systolic blood pressure (continuous), physical activity, diabetes, insulin resistance (top quartile vs others), C-reactive protein, high-density lipoprotein cholesterol, and triglycerides (continuous).

d Model 3 = Model 2 + impaired cerebrovascular reactivity vs normal, carotid intima-media thickness, global cognitive score, and geriatric despression score ≥5 at time 1 (2004–2009).

e Model 4 = Model 3 applying inverse probability weights.

f Model 5 = Model 3 after excluding 53 participants with stroke and dementia at time 2 (2011–2013).

## Discussion

In this cross-sectional study of men with CVD, being overweight and obese in their fifties was related to late-life sarcopenia. The observed relationship was independent of traditional cardiovascular risk factors and global cognitive score and did not change after exclusion of participants with a history of stroke or dementia at the sarcopenia assessment. To the best of our knowledge, our study is the first to estimate the association between obesity and overweight and late-life sarcopenia among men with CVD. In our sample, the prevalence of probable sarcopenia (33.2%) and sarcopenia (34.4%) was high compared with the prevalence in the general population (5%–13%) measured by using the criteria of the European Working Group on Sarcopenia in Older People (2). A retrospective study of Japanese men with CVD and an average age of 72 (SD, 12) found sarcopenia in 29.5% of the study population (30). The prevalence of sarcopenia is higher among people with CVD than among the general population, but comparisons of sarcopenia rates between studies are difficult because of differences in study population, age ranges, sarcopenia definition, and the use of various thresholds for defining low muscle mass and low muscle performance (10,31).

Some researchers have suggested that being overweight in midlife may be associated with lower, rather than higher, sarcopenia rates (32). In a 4-year follow-up study among community-dwelling Chinese men and women aged 72.5 (SD, 5.2) on average found that higher BMI was inversely associated with the development of sarcopenia (7). On the other hand, in the Collaborative Research on Ageing in Europe survey conducted among people aged 65 or older, higher percentage body fat and lower levels of physical activity were associated with low muscle mass and sarcopenia in almost all the countries studied (10).

Obesity is associated with increased risk of glucose intolerance, chronic inflammation, hypertension, dyslipidemia, and CVD. Sarcopenia shares many pathological mechanisms with obesity, including insulin resistance and low-grade chronic inflammation (3,5). Aging induces changes in body composition, such as increases in visceral fat and decreases in skeletal muscle mass (10). Fat mass induces inflammation, which may contribute to the development of sarcopenia (33). A vicious circle between muscle loss and fat gain may lead to more sarcopenia and then to further weight gain, inflammation, and impaired glucose tolerance (5). However, these changes in body composition, such as increases in fat and decreases in muscle mass, are potentially reversible by modifying lifestyle behaviors (7,33). Resistance exercise and nutrition support, which increases muscle mass and muscle strength, are key lifestyle strategies that can prevent sarcopenia or reverse it (34).

Strengths of this study include a unique data set of extensively studied men with CVD who underwent comprehensive evaluations. Skeletal muscle mass was assessed by bioimpedance method, which is considered simple and inexpensive and has a high correlation with magnetic resonance imaging and dual-energy x-ray absorptiometry (19). However, some important issues need to be considered. First, we could not evaluate the causal effect of obesity on sarcopenia because we did not assess sarcopenia at baseline. Second, the generalizability of the study is limited to men with CVD who had the specific clinical characteristics required for eligibility in the BIP study. Finally, despite an acceptable response rate of 82% among survivors, a substantial proportion of patients did not participate in the late-life assessment because of the long period between baseline and the late-life evaluations.

In summary, we observed a positive association between overweight and obese men with CVD and late-life sarcopenia. Given the growth of the population aged 65 or older and the high prevalence of obesity and CVD (11,12), it is important to identify people with CVD and potentially preventable risk factors for sarcopenia. However, these findings need to be confirmed in larger studies.

## Post-Test Information

To obtain credit, you should first read the journal article. After reading the article, you should be able to answer the following, related, multiple-choice questions. To complete the questions (with a minimum 75% passing score) and earn continuing medical education (CME) credit, please go to http://www.medscape.org/journal/pcd. Credit cannot be obtained for tests completed on paper, although you may use the worksheet below to keep a record of your answers.

You must be a registered user on http://www.medscape.org. If you are not registered on http://www.medscape.org, please click on the “Register” link on the right hand side of the website.

Only one answer is correct for each question. Once you successfully answer all post-test questions, you will be able to view and/or print your certificate. For questions regarding this activity, contact the accredited provider, CME@medscape.net. For technical assistance, contact CME@medscape.net. American Medical Association’s Physician’s Recognition Award (AMA PRA) credits are accepted in the US as evidence of participation in CME activities. For further information on this award, please go to https://www.ama-assn.org. The AMA has determined that physicians not licensed in the US who participate in this CME activity are eligible for AMA PRA Category 1 Credits™. Through agreements that the AMA has made with agencies in some countries, AMA PRA credit may be acceptable as evidence of participation in CME activities. If you are not licensed in the US, please complete the questions online, print the AMA PRA CME credit certificate, and present it to your national medical association for review.

## Post-Test Questions

### Study Title: Overweight, Obesity, and Late-Life Sarcopenia Among Men With Cardiovascular Disease, Israel

#### CME Questions

1. Your patient is a 57-year-old obese man with early evidence of sarcopenia. According to the study by Lutski and colleagues, which of the following statements about the prevalence of sarcopenia and associated factors among community-dwelling elderly men with cardiovascular disease (CVD) is correct?
  - Fewer than one-quarter of the total sample had sarcopenia
  - One-third of patients with obesity (body mass index [BMI] ≥ 30 kg/m^2^) and one-quarter of patients with overweight (BMI 25 to ≤ 29.9 kg/m^2^) had sarcopenia
  - Sarcopenia was not significantly associated with baseline systolic blood pressure (SBP)
  - Patients with vs without sarcopenia had poorer global cognitive function
2. According to the study by Lutski and colleagues, which of the following statements about the increased risk for sarcopenia as a function of BMI among community-dwelling elderly men with CVD is correct?
  - Comparing BMI ≥ 25 to BMI < 25 kg/m^2^, adjusted odds ratio (aOR) for probable sarcopenia was 3.27 (95% CI: 1.68, 6.36) and for sarcopenia was 5.31 (95% CI: 2.5, 11.27)
  - Further adjustment for age, education, birthplace, SBP, physical activity, insulin resistance, CRP, high-density lipoprotein cholesterol (HDL-C), and triglycerides abolished the association of sarcopenia with BMI
  - Further adjustment for global cognitive score and ultrasound-doppler indices of cerebrovascular disease and depressive symptoms strengthened the association of sarcopenia with BMI
  - For each 1-kg/m^2^ increase in BMI, risk for sarcopenia increase 5%
3. According to the study by Lutski and colleagues, which of the following statements about clinical and public health implications of the association among overweight, obesity, and late-life sarcopenia among community-dwelling elderly men with CVD is correct?
  - The study proved that obesity causes sarcopenia
  - The association of obesity with late-life sarcopenia suggested that obesity may be an important modifiable factor related to sarcopenia among men with CVD
  - Pathologic mechanisms in sarcopenia do not overlap with those in obesity
  - Aerobic exercise is the best known intervention to prevent or decrease sarcopenia
